# Functional divergence of the sarcomeric myosin, MYH7b, supports species-specific biological roles

**DOI:** 10.1016/j.jbc.2022.102657

**Published:** 2022-11-09

**Authors:** Lindsey A. Lee, Samantha K. Barrick, Artur Meller, Jonathan Walklate, Jeffrey M. Lotthammer, Jian Wei Tay, W. Tom Stump, Gregory Bowman, Michael A. Geeves, Michael J. Greenberg, Leslie A. Leinwand

**Affiliations:** 1 Molecular, Cellular, and Developmental Biology Department, Boulder, Colorado, USA; 2 BioFrontiers Institute, University of Colorado Boulder, Boulder, Colorado, USA; 3 Department of Biochemistry and Molecular Biophysics, Washington University School of Medicine, St Louis, Missouri, USA; 4 The Center for Science and Engineering of Living Systems, Washington University in St Louis, St Louis, Missouri, USA; 5 School of Biosciences, University of Kent, Canterbury, United Kingdom; 6 Department of Biochemistry and Biophysics, University of Pennsylvania, Philadelphia, Pennsylvania, USA

**Keywords:** myosin, actin, structure–function, cardiac muscle, skeletal muscle, kinetics, molecular motor, super-relaxed state, interacting heads motif, BSA, bovine serum albumin, cDNA, complementary DNA, CMV, cytomegalo virus, DRX, disordered-relaxed state, EOM, extraocular muscle, HEK293, human embryonic kidney 293 cell line, IHM, interacting heads motif, MSM, Markov state model, MYH7b, myosin heavy chain 7b, MyHC, myosin heavy chain, S1, subfragment 1, SRX, super-relaxed state

## Abstract

Myosin heavy chain 7b (MYH7b) is an evolutionarily ancient member of the sarcomeric myosin family, which typically supports striated muscle function. However, in mammals, alternative splicing prevents MYH7b protein production in cardiac and most skeletal muscles and limits expression to a subset of specialized muscles and certain nonmuscle environments. In contrast, MYH7b protein is abundant in python cardiac and skeletal muscles. Although the MYH7b expression pattern diverges in mammals *versus* reptiles, MYH7b shares high sequence identity across species. So, it remains unclear how mammalian MYH7b function may differ from that of other sarcomeric myosins and whether human and python MYH7b motor functions diverge as their expression patterns suggest. Thus, we generated recombinant human and python MYH7b protein and measured their motor properties to investigate any species-specific differences in activity. Our results reveal that despite having similar working strokes, the MYH7b isoforms have slower actin-activated ATPase cycles and actin sliding velocities than human cardiac β-MyHC. Furthermore, python MYH7b is tuned to have slower motor activity than human MYH7b because of slower kinetics of the chemomechanical cycle. We found that the MYH7b isoforms adopt a higher proportion of myosin heads in the ultraslow, super-relaxed state compared with human cardiac β-MyHC. These findings are supported by molecular dynamics simulations that predict MYH7b preferentially occupies myosin active site conformations similar to those observed in the structurally inactive state. Together, these results suggest that MYH7b is specialized for slow and energy-conserving motor activity and that differential tuning of MYH7b orthologs contributes to species-specific biological roles.

Members of the sarcomeric myosin heavy chain (MyHC) family function as actin-based motors in striated muscle, where they convert chemical energy from ATP hydrolysis into the mechanical force necessary for contraction. Studies have focused almost exclusively on the eight conventional sarcomeric myosin-II genes principally expressed in the major skeletal muscles and the heart (*MYH1*, *MYH2*, *MYH3*, *MYH4*, *MYH6*, *MYH7*, *MYH8*, and *MYH13*). These sarcomeric myosin proteins consist of a globular motor domain with ATP and actin-binding sites, a neck domain bound by myosin light chains, and an ɑ-helical tail domain that drives assembly into the thick filament of the sarcomere. Despite high sequence conservation across this myosin protein family, the properties of the chemomechanical cycle differ greatly among isoforms allowing for specific tuning of muscle physiology (1, 2, 3).

Given the deep scrutiny of the sarcomeric myosin family because of its essential role in life-critical organs, it was surprising to discover additional members of this family, including MYH7b, after bioinformatic annotation of the human genome (4). This oversight was the result of the atypical expression pattern of MYH7b in mammals. *MYH7b* transcripts are present in mammalian cardiac and major skeletal muscles, but a regulated alternative splicing event introduces a premature stop codon that prevents MYH7b protein production in these tissues (5). Moreover, forced cardiac expression of MYH7b protein in a transgenic mouse model resulted in a severe dilated cardiomyopathy, underscoring the importance of evolutionary silencing of MYH7b protein production in the mammalian heart (6). However, MYH7b protein is found in the sarcomeres of certain mammalian specialized muscles, such as extraocular muscles (EOMs), muscle spindles, and the upper esophagus (7, 8). Surprisingly, MYH7b protein has also been detected at low abundance in nonmuscle tissues, and mutations in MYH7b are linked to hereditary hearing loss (5, 9, 10). Given this unique expression pattern, it remains unclear how MYH7b functions in certain mammalian specialized muscles and nonmuscle tissue, yet is not well tolerated in major muscles like the heart.

Phylogenetic analysis of the sarcomeric myosin family classifies MYH7b as an ancient myosin that predates the emergence of the major cardiac and skeletal myosin isoforms (4). Immunostaining has identified MYH7b protein in chicken heart and skeletal muscle (7, 11), and in this study, we report MYH7b protein identification in python heart and skeletal muscles. Given the difference in expression pattern of MYH7b between reptiles/birds and mammals, it appears that the MYH7b gene has evolved to meet different physiological needs of these distinct vertebrate species. Based on this divergent expression pattern across species and the ancient evolutionary roots of MYH7b, we hypothesized that (1) human MYH7b motor properties diverge from other well-characterized human sarcomeric myosin family members and (2) human and python MYH7b motor functions are differentially tuned to accommodate species-specific roles. We examined these hypotheses using a comprehensive suite of biochemical and biophysical analyses to test MYH7b motor activity. Ultimately, our study of MYH7b defines the activity of a previously uncharacterized sarcomeric myosin motor and provides an evolutionary perspective of motor function within this highly conserved protein family.

## Results

### MYH7b is detected at the RNA and protein levels in python muscles

**Figure 1:**
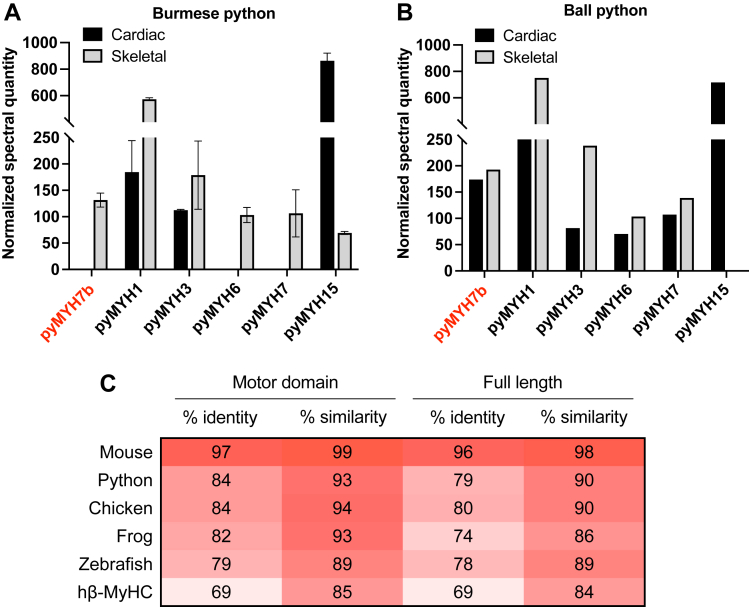
**Mass spectrometry detects MYH7b protein in python muscle.** Normalized spectral quantity of python myosin heavy chain proteins detected in cardiac and skeletal samples of two distinct python species, *A*, Burmese python (n = 2) and (*B*) Ball python (n = 1). MYH7b is present in skeletal muscle of Burmese pythons and the cardiac and skeletal muscle of Ball pythons. Note that all mass spectrometry values are semiquantitative as these experiments do not include labeled internal standards. The mass spectrometry data are summarized in Tables S1 and S2. Data are graphed as mean ± SD. *C*, percent identity and sequence similarity to human MYH7b calculated from sequence alignments using NCBI BLAST of MYH7b protein sequence across species. NCBI accession codes are as follows: human NP_065935.4, mouse NP_001078847.1, python XP_007419944.1, chicken NP_989918.3, frog XP_031750297.1, and zebrafish NP_001311408.1. MYH7b, myosin heavy chain 7b; NCBI, National Center for Biotechnology Information.

Most mammalian muscle tissues, including the heart and major skeletal muscles, do not produce MYH7b protein because of an alternative splicing event that shifts a premature stop codon into frame (5, 12). We investigated whether the MYH7b expression pattern observed in mammals is conserved in reptiles, an evolutionarily distinct class of vertebrates, by examining the cardiac and skeletal muscle myosin composition of two distinct species of python, Burmese python (*Python bivittatus*) and Ball python (*Python regius*). We detected *MYH7b* RNA and unique MYH7b peptides in the skeletal muscle of both python species and in the cardiac muscle of Ball pythons but not Burmese pythons (Figs. 1 and S1). Notably, pythons express a broad array of myosins in their muscles where MYH15 (the other recently identified ancient myosin gene) appears to be the predominant cardiac myosin isoform, and MYH1 is the most abundant skeletal myosin isoform (Fig. 1, *A*, *B*, Tables S1 and S2). We also assessed the myosin light chain composition in these tissues to understand which myosin light chains are present (Fig. S1, *A*–*C*). All python myosin light chain sequences share 69 to 99% sequence identity with their mouse myosin light chain orthologs. Using an RT–PCR assay that discriminates between the two *MYH7b* splice forms, we determined that the alternative splicing event first discovered in mammals (5) also prevents MYH7b protein production in Burmese python hearts (Fig. S1*E*). Ball python cardiac *MYH7b* RNA also undergoes exon skipping, but a proportion of unskipped RNA that can encode protein is readily detectable (Fig. S1*E*). Based on these results, it appears that, unlike the mammalian ortholog, MYH7b operates in a conventional role in python cardiac and skeletal muscle.

Despite the divergent expression pattern observed in mammals *versus* reptiles, the MYH7b sequence is highly conserved across species (Fig. 1*C*). Therefore, it is unclear whether inherent molecular function diverges between human and python MYH7b or whether cell type–specific factors within the different tissues that express MYH7b externally tune MYH7b properties to promote species-specific roles. To directly compare human and python MYH7b motor properties, we produced recombinant myosin motor domains consisting of the myosin subfragment 1 (S1) region (Fig. S2) and performed a comprehensive analysis of their catalytic motor properties. Our studies use human β-MyHC (encoded by the *MYH7* gene), the myosin isoform expressed in cardiac and slow skeletal muscle, as a comparison because β-MyHC is the closest in sequence identity to human MYH7b and has been extensively characterized *in vitro* (2, 13).

### Human and python MYH7b have slower actin-activated ATPase activity than human β-MyHC

**Figure 2:**
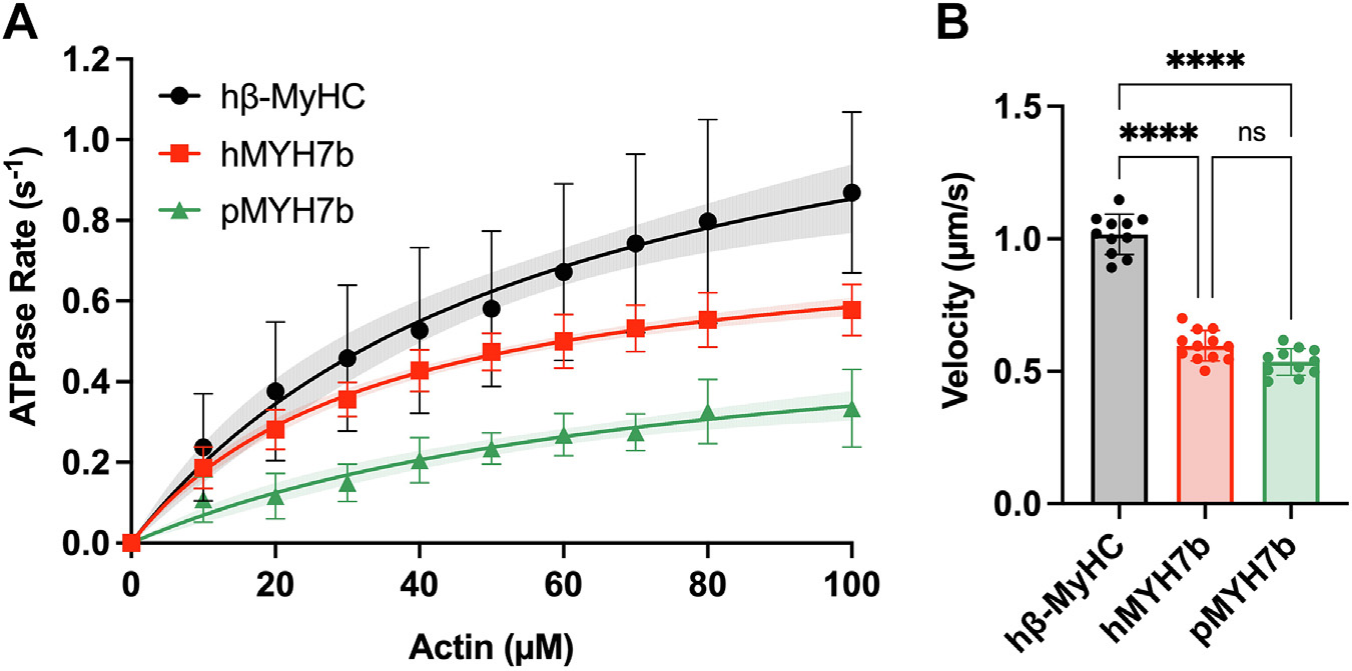
**Human and python MYH7b have slow motor properties.***A*, actin-activated ATPase curves of human β-MyHC S1, human MYH7b S1, and python MYH7b S1. Each plot shows the average of all technical replicates, and error bars represent SD. Data were fit to a Michaelis–Menten kinetics equation to obtain the *k*_cat_ and *K*_*M*_ values summarized in Table S4. The curve fit is represented by a *solid line* with shading to indicate the 95% confidence intervals. The average of ATPase curves run on the same day represent technical replicates (hβ-MyHC [n = 9], hMYH7b [n = 9], and pMYH7b [n = 5]), and at least four different purifications (biological replicates) are represented for each construct. *B*, *in vitro* motility velocities of human β-MyHC S1, human MYH7b S1, and python MYH7b S1. The average velocity of each technical replicate (each motility video) is graphed with error bars representing SD. Data were collected for at least three independent protein purifications (biological replicates). Technical replicates: hβ-MyHC (n = 11), hMYH7b (n = 12), and pMYH7b (n = 11). Data are summarized in Table S5. ∗∗∗∗ indicates *p* < 0.0001. MYH7b, myosin heavy chain 7b; β-MyHC, beta myosin heavy chain; S1, subfragment 1.

We used an NADH-coupled system to assess the steady-state actin-activated ATPase activity of our myosin constructs. We measured the ATPase rates of human β-MyHC, human MYH7b, and python MYH7b at increasing actin concentrations and fit the Michaelis–Menten kinetics equation to the data to calculate the maximum actin-activated ATPase rate (per myosin molecule, *k*_cat_) and apparent actin affinity (*K*_*M*_) for each myosin construct (Fig. 2*A* and Table S4). Human MYH7b has a maximal ATPase rate of 0.80 ± 0.08 s^−1^, strikingly lower by 49% compared with the canonical slow human β-MyHC isoform (1.57 ± 0.18 s^−1^, *p* < 0.0001). The maximal ATPase rate for python MYH7b (0.60 ± 0.08 s^−1^) was substantially lower than both β-MyHC (a 62% decrease, *p* < 0.0001) and human MYH7b (a 25% decrease, *p* = 0.0350). The maximum actin-activated ATPase rate is inversely proportional to the total cycle time (*t*_cycle_) through the chemomechanical cycle for one myosin (*k*_cat_ = 1/*t*_cycle_) (14). Thus, when comparing *t*_cycle_ between isoforms from the values measured, β-MyHC has the shortest cycle time (∼0.6 s) and human and python MYH7b have a longer *t*_cycle_ comparatively (∼1.3 s and ∼1.7 s, respectively). Moreover, the *K*_*M*_ of human MYH7b (35.9 ± 9.8 μM) is 62% lower than human β-MyHC (93.4 ± 56.5 μM, *p* = 0.0096), indicating that MYH7b binds more tightly to actin. The *K*_*M*_ of python MYH7b (79.3 ± 16.1 μM) is not significantly different than that of human β-MyHC indicating that altered actin affinity cannot account for the decreased *k*_cat_ for python MYH7b. Finally, we assessed overall enzymatic efficiency of actin-activated ATPase for each construct determined by *k*_cat_/*K*_*M*_. We observed similar measures for human β-MyHC (0.025 ± 0.015 s^−1^ μM^−1^) and human MYH7b (0.023 ± 0.006 s^−1^ μM^−1^), whereas comparatively python MYH7b is a less efficient motor (0.008 ± 0.003 s^−1^ μM^−1^).

### Human and python MYH7b actin sliding velocities do not differ from each other but are slower than human β-MyHC

In order to understand the mechanical properties of MYH7b, we used an unloaded *in vitro* motility assay to determine the actin sliding velocities of human and python MYH7b compared with human β-MyHC (Fig. 2*B*, Table S5, and Movies S1–S3). For these experiments, we used PDZ-based chemistry to immobilize our C-terminally tagged S1 constructs to a coverslip and monitored the velocity at which each myosin moved fluorescently labeled actin. Experiments measuring the actin sliding velocity as a function of myosin concentration were conducted to ensure surface saturation (Fig. S3). Human MYH7b and python MYH7b actin sliding velocities (0.597 ± 0.057 μm/s and 0.536 ± 0.076 μm/s, respectively) were lower compared with human β-MyHC (1.017 ± 0.050 μm/s, *p* < 0.0001 for both). There was no discernable difference between human and python MYH7b actin sliding velocities.

### Stopped-flow experiments reveal that human and python MYH7b have slow kinetics

**Figure 3:**
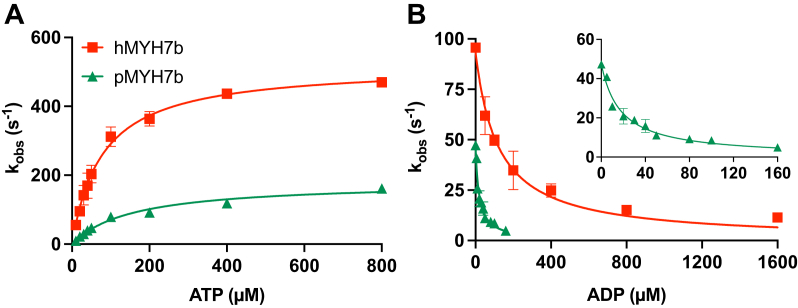
**Human and python MYH7b have distinct kinetic properties.***A*, ATP-induced dissociation of myosin S1 from actin plotted as observed rate constants against ATP concentration. The python *k*_obs_ values were lower than the human values at all ATP concentrations. The *k*_obs_*versus* ATP concentration data were fit to a hyperbolic equation and yield values of *k*_+2_ and 1/*K*_1_ as described in the Experimental procedures section. *B*, myosin S1 affinity for ADP. As ADP concentration increases, the observed rate constants decrease and can be fit to a hyperbolic equation. The reaction rate constants were slower for python MYH7b (see *inset*) at all ADP concentrations than for human MYH7b, and analysis of the curve indicates that human MYH7b has a weaker affinity for ADP. Final ATP concentrations were 20 μM for human MYH7b and 50 μM for python MYH7b. Data represent mean ± SD, n = 2 for each experiment; values and statistics are summarized in Table S6. MYH7b, myosin heavy chain 7b.

We next performed stopped-flow experiments to better understand the kinetics of key steps of the chemomechanical cycle that may underly the differences in actin-activated ATPase activity observed between human MYH7b and python MYH7b. The dissociation of human and python MYH7b from pyrene-labeled actin was best described by a single exponential, which provides an observed rate constant (*k*_obs_). The rates of ATP-induced dissociation of myosin from actin are plotted against ATP concentration in Figure 3*A* (stopped-flow data are summarized in Table S6). A linear dependence at low ATP concentrations provides the second-order rate constant for ATP binding (*K*_1_*k*_+2_, Fig. S4. The ATP-binding rate for human MYH7b was faster (6.7 ± 1.4 μM^−1^ s^−1^) than the rate previously measured for human β-MyHC S1 (∼4.4 μM^−1^ s^−1^) (15), whereas python MYH7b had a slower rate than both (1.2 ± 0.0 μM^−1^ s^−1^). Over the full range of ATP concentrations, the data are described by a hyperbolic fit (Equation 1), which results in the maximum dissociation rate (*k*_+2_) and the ATP-binding affinity in the initial step (1/*K*_1_). Both human MYH7b (79.8 ± 17.7 μM) and python MYH7b (157.3 ± 11.5 μM) had a much tighter affinity for ATP (smaller 1/*K*_1_) compared with values for human β-MyHC (∼365.7 μM) (15). However, human MYH7b ATP affinity was twofold tighter than that of python MYH7b (*p* = 0.0352). Similarly, human MYH7b (520.7 ± 7.0 s^−1^) and python MYH7b (181.2 ± 6.9 s^−1^) have a slower maximum actin dissociation rate compared with human β-MyHC (∼991 s^−1^). It is worth noting that the dissociation rate for python MYH7b is over 2.5-fold slower than human MYH7b (*p* = 0.0004).

We next determined the ADP affinity for each myosin S1 construct using a competition assay where increasing concentrations of ADP were rapidly mixed alongside a fixed ATP concentration with S1, resulting in a competition between the ATP and ADP for the nucleotide-binding site of myosin (Fig. 3*B*). As the concentration of ADP increases, the *k*_obs_ decreases as more ADP is available to bind myosin. Plotting the *k*_obs_ against the ADP concentration can be described by a hyperbolic fit (Equation 2), which provides a measure of the ADP affinity (*K*_ADP_). Human MYH7b had a weaker ADP affinity (120.7 ± 29.5 μM) compared with both python MYH7b (17.0 ± 3.2 μM, *p* = 0.0386) and human β-MyHC (∼6.1 μM) (15). Taken together, these results indicate that human and python MYH7b have appreciably slower kinetics than human β-MyHC.

### Human and python MYH7b have similar step sizes but slower detachment kinetics than human β-MyHC

**Figure 4:**
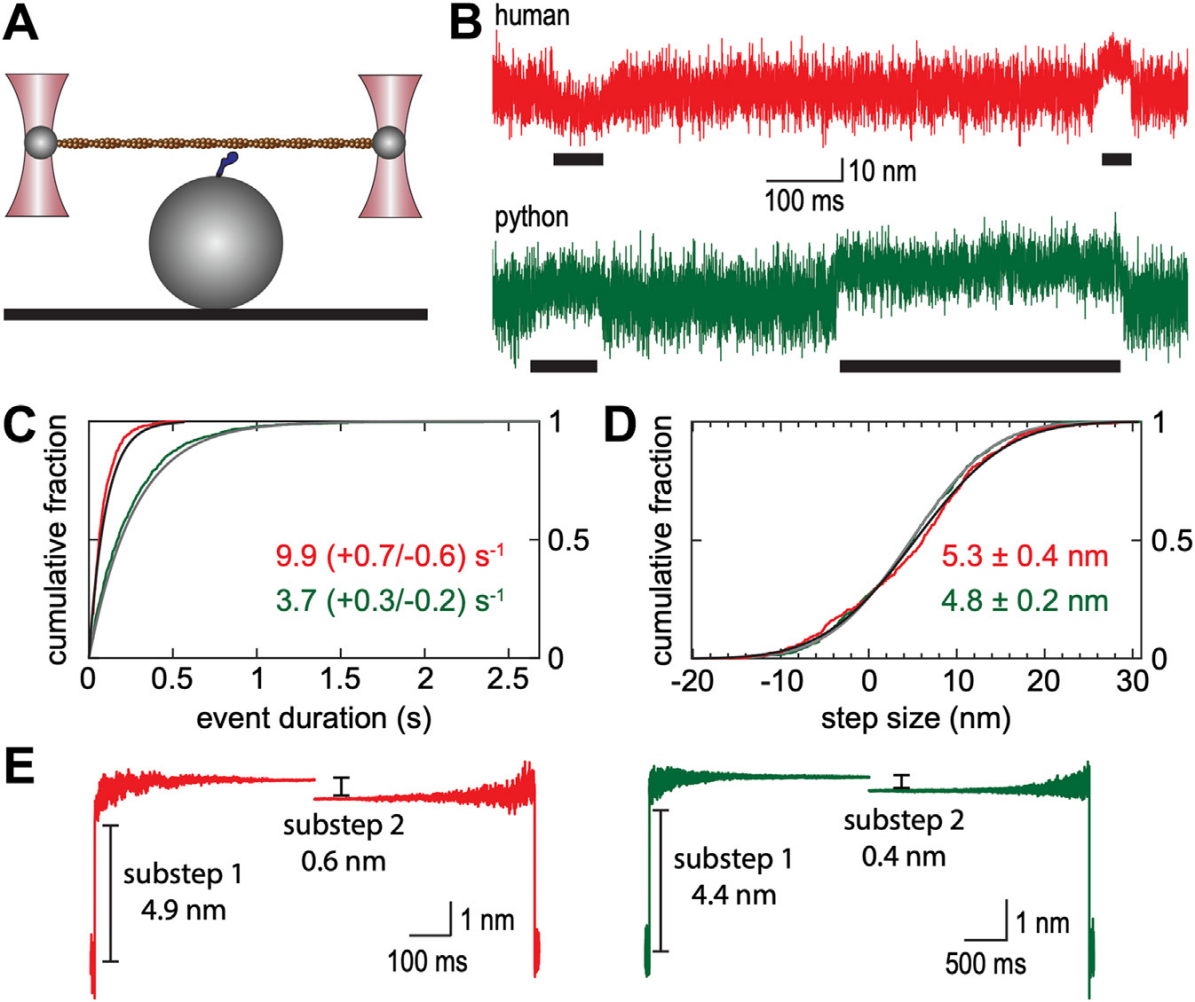
**Mechanical and kinetic characterization of human and python MYH7b by optical trapping.***A*, schematic of three-bead optical trapping assay in which an actin filament is suspended between two optically trapped beads and lowered on to a surface bead that is sparsely coated with myosin. *B*, sample data traces for human MYH7b S1 (*red*) and python MYH7b S1 (*green*). Binding events, which are associated with reduced variance of the bead position and a shift in the mean bead position, are marked by *black bars*. *C*, cumulative distribution of binding interaction durations observed for human MYH7b S1 (*red data trace*, *black fit line*) and python MYH7b S1 (*green data trace*, *gray fit line*) at 1 μM ATP. The detachment rate of python MYH7b is faster than the rate for human MYH7b (*p* < 0.001). *D*, cumulative distribution of total step sizes observed for human MYH7b S1 (*red data trace*, *black fit line*) and python MYH7b S1 (*green data trace*, *gray fit line*). Step sizes are reported as mean ± SEM. The total working strokes of python and human MYH7b are not significantly different (*p* = 0.27). *E*, ensemble averages of individual binding events for human MYH7b S1 (*left*, *red*) and python MYH7b S1 (*right*, *green*) showing a two-substep working stroke. MYH7b, myosin heavy chain 7b; S1, subfragment 1.

We next sought to determine the changes in motor function that contribute to the slower actin sliding velocities of the MYH7b constructs compared with β-MyHC. The speed in the motility assay at saturating myosin concentrations is set by the displacement of the myosin working stroke (d) and the amount of time that myosin spends attached to actin at saturating ATP (*t*_on_). We used a single-molecule optical trapping assay (Fig. 4*A*) based on the three-bead geometry (16) to define the size of the working stroke for human and python MYH7b (17, 18). In this assay, a flow cell is coated with surface beads that are sparsely decorated with myosin S1. Within the flow cell, an actin filament is suspended between two optically trapped beads and brought close to a surface bead to probe for interactions with myosin S1. Actomyosin-binding interactions are associated with decreased variance of trapped bead positions and a shift in the mean bead positions, the magnitude of which reports on the step size of myosin S1 undergoing its working stroke (Fig. 4*B*). The distribution of attachment durations can be used to calculate the rate of dissociation of myosin from actin. These experiments are conducted at low ATP concentrations to facilitate the observation of binding interactions.

We measured the detachment kinetics of human and python MYH7b S1 at subsaturating ATP concentrations (1 μM ATP). Under these conditions, the rate of actomyosin dissociation is limited by the rate of ATP binding, not ADP release as seen in the motility assays. We found that the detachment rate for python MYH7b (3.7 [+0.3/−0.2] s^−1^) was slower than the detachment rate for human MYH7b (9.9 [+0.7/−0.6] s^−1^; *p* < 0.001) (Fig. 4, *B* and *C*). This demonstrates that the rate of ATP-induced actomyosin dissociation is slower for python relative to human MYH7b, consistent with the second-order rates of ATP-induced dissociation at low ATP measured in the stopped-flow (*K*_1_*k*_+2_). Interestingly, the detachment rate for python MYH7b, which is expressed in python hearts, is similar to that previously observed for human β-MyHC myosin (∼3 s^−1^) (19). These results demonstrate a biophysical difference between human and python MYH7b.

We also measured the size of the myosin working stroke. We observed similar total step sizes for human MYH7b (5.3 ± 0.4 nm) and python MYH7b (4.8 ± 0.2 nm; *p* = 0.27) (Fig. 4*D*). These step sizes are consistent with those previously observed for human and porcine β-cardiac myosin (∼5–7 nm) (2, 15, 18, 19, 20, 21). Next, we examined whether there are substeps to the MYH7b working stroke. Many myosin isoforms, including β-MyHC myosin, have a two-substep working stroke (18, 22, 23, 24, 25, 26), which can be resolved using ensemble averaging of individual binding events (17, 27). Ensemble averaging involves postsynchronization of binding events and averaging forward in time from actomyosin attachment (time-forward average) or backward in time from actomyosin detachment (time-reversed average). If the working stroke consists of multiple substeps, there will be a difference in displacement between the time-forward and time-reversed averages equal to the size of the second displacement. Our data clearly demonstrate a two-substep working stroke for both human and python MYH7b (Fig. 4*E*), demonstrating that the mechanics of the MYH7b working stroke are similar to β-MyHC. Taken together, our results demonstrate that the reduced velocity in the motility assay is because of changes in the myosin kinetics and not mechanics.

### Human MYH7b and python MYH7b display differences in super-relaxed state/disordered-relaxed state proportions

**Figure 5:**
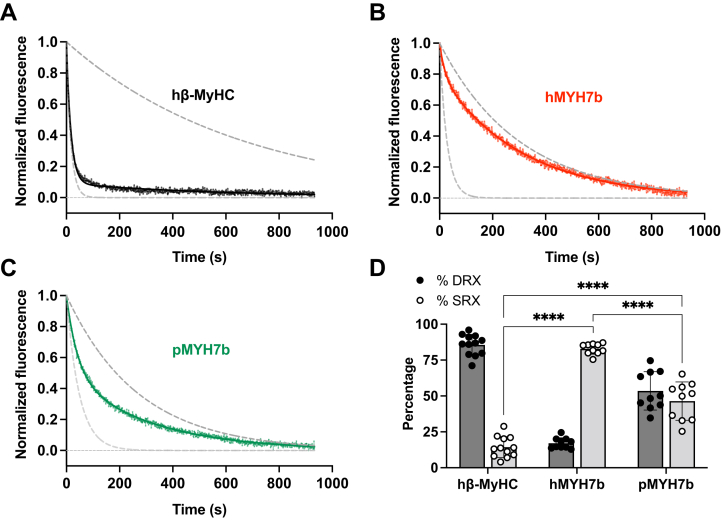
**Human and python MYH7b have different proportions of SRX populations.***A*–*C*, representative traces for single mant-ATP turnover experiments with human β-MyHC S1 (*A*), human MYH7b S1 (*B*), and python MYH7b S1 (*C*). Each normalized curve was fit to a biexponential decay with Y_0_ = 1 and plateau = 0. The *top dark gray dotted line* represents data simulated with a single exponential decay with the average slow rate for each construct, and the *bottom light gray dotted line* represents data simulated with a single exponential decay with the average fast rate for each construct. *D*, quantification of the percent SRX and DRX for each construct. Data points represent technical replicates (individual curves) comprised of at least four separate purifications (biological replicates), and error bars represent SD. Technical replicates: hβ-MyHC (n = 12), hMYH7b (n = 10), and pMYH7b (n = 10). ∗∗∗∗ indicates *p* < 0.0001. β-MyHC, beta myosin heavy chain; DRX, disordered-relaxed state; mant, 2'-(or-3')-*O*-(*N*-methylanthraniloyl); MYH7b, myosin heavy chain 7b; S1, subfragment 1; SRX, super-relaxed state.

Muscle function and energy usage by myosin can be modulated by shifting the equilibrium of myosin into different functional states. Myosin ATPase activity is fastest when actomyosin crossbridge cycling occurs, but ATP turnover decreases when myosin is unavailable to bind actin. Non–actin-bound myosin can adopt two distinct functional states: the disordered-relaxed state (DRX; 100-fold slower than actin-activated rates) or the super-relaxed state (SRX; ultralow ATPase, 1000-fold slower than actin-activated rates) (28, 29, 30, 31). Based on the low actin-activated ATPase activity we observed for MYH7b, we hypothesized that the ratio of SRX/DRX for MYH7b may inherently shift toward SRX compared to β-MyHC and alter the number of myosin motors available to enter the actin-bound state. We used the 2'-(or-3')-*O*-(*N*-methylanthraniloyl)-ATP (mant-ATP) single turnover kinetics assay to measure the population of myosin motor domains in the SRX and DRX states and the associated enzymatic rates of the myosin S1 constructs in the absence of actin (Fig. 5 and Table S7). We observed that human MYH7b has a substantially higher proportion of myosin heads in the SRX state as compared with human β-MyHC (82.9 ± 3.7% *versus* 14.4 ± 7.3%, respectively; *p* < 0.0001). Intriguingly, python MYH7b has a lower proportion of myosin heads in the SRX state compared with human MYH7b (46.4 ± 13.3%, *p* < 0.0001) but still a considerably higher proportion of SRX state myosin compared with human β-MyHC (*p* < 0.0001). The DRX ATP turnover rates were consistent with the actin-activated ATPase rates calculated for each construct where human β-MyHC was faster than human MYH7b and python MYH7b had the slowest rate (Table S7). These assays were performed with single-headed myosin S1, and our measurements are consistent with previous measurements for human β-MyHC short S1 constructs where the slower ATPase rate is thought to arise from specific motor domain and lever arm conformations (28, 29, 30, 32). These data suggest that both MYH7b constructs shift toward a more energy-conserving state.

### MYH7b is more likely to adopt closed switch-2 conformations

**Figure 6:**
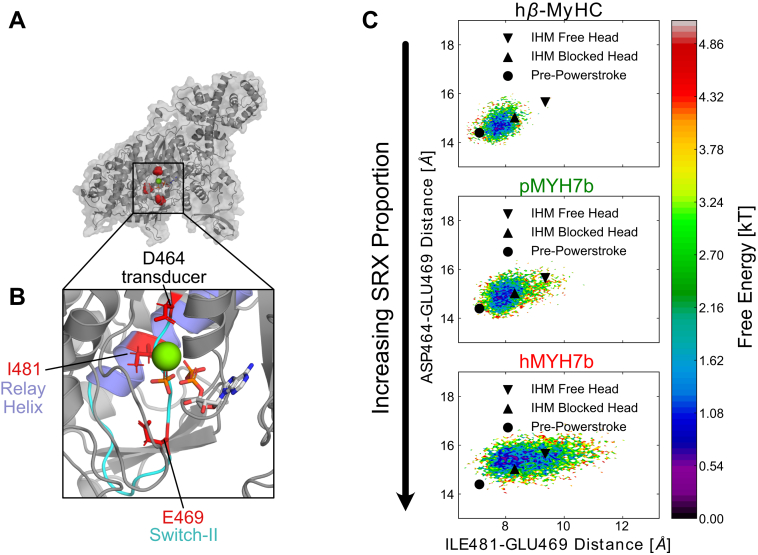
**Molecular dynamics simulations predict that human MYH7b is more likely to adopt closed switch-2 conformations similar to those observed in the interacting heads motif (IHM) active site.***A*, homology model of human MYH7b S1 in the pre-powerstroke state. *Red spheres* indicate key active site residues whose structural fluctuations differ between MYH7b and β-MyHC. The active site ligands (ADP∗Pi∗Mg) are aligned and superimposed as reference. *B*, zoom-in of the human MYH7b active site. The three residues (D464, E469, and I481) depicted in *A* are shown in *red sticks*. The relay helix is shown in *blue*, whereas switch-2 is shown in *cyan*. The active site ligands (ADP∗Pi∗Mg) are aligned and superimposed as reference. *C*, free energy landscapes of switch-2 E469’s position show that E469 is more likely to occupy positions farther away from the relay helix and transducer in MYH7b, more similar to the interacting heads motif free head structure. *Black circle* indicates the position of E469 in the pre-powerstroke crystal structure that was used as the starting point for simulations (Protein Data Bank code: 5N6A), whereas the *black triangles* indicate the position of E469 in the interacting heads motif (upward facing for the blocked head and downward facing for the free head). β-MyHC, beta myosin heavy chain; MYH7b, myosin heavy chain 7b; S1, subfragment 1.

We next used molecular dynamics simulations to investigate the structural differences between human and python MYH7b and human β-MyHC that may give rise to the large shift in SRX/DRX proportions seen in the ATP turnover assay. At present, there are no available experimental structures of MYH7b. Therefore, we homology modeled the MYH7b sequence into a β-MyHC S1 structural template (Protein Data Bank code: 5N6A) (33) to perform simulations (Fig. 6*A*). Though human MYH7b, python MYH7b, and human β-MyHC simulations were initiated from the same starting structure, previous molecular dynamics simulations revealed conformational heterogeneity missing in ground state structural models (34). Based on our SRX measurements, we hypothesized that human MYH7b, and to a lesser extent, python MYH7b, would shift the distribution of conformations adopted in the active site toward inactive states with slow basal ATPase activity. A recently published cryo-electron microscopy structure of the interacting heads motif (IHM) reveals a novel active site geometry that may be crucial for achieving the SRX biochemical state (35). Importantly, in the IHM structure, switch-2, an active site loop that coordinates phosphate, is shifted away from the relay helix into a putative phosphate release pathway (Fig. 6*C*). We reasoned that MYH7b would be more likely to adopt similar active site conformations to those observed in the IHM.

To find differences between the structural ensembles of MYH7b and β-MyHC, we used a self-supervised autoencoder called DiffNets (36). Subsequently, we measured the probabilities of these structural changes occurring between isoforms by combining hundreds of microseconds of simulation data using Markov state models (MSMs) (37). Briefly, DiffNets learns a low-dimensional representation of each simulation frame (*i.e.*, a latent vector) and outputs a classification label for each frame in a self-supervised manner based on the expectation maximization algorithm. The DiffNet assigns each frame a probability from 0 to 1 that represents the likelihood of an active site conformation being unique to the MYH7b structural ensemble. By finding the correlation between structural features and this output label, we can identify structural differences that discriminate MYH7b from β-MyHC (Fig. S5). To quantify these differences, we constructed MSMs of the entire S1 construct (see Experimental Procedures section) and compared the equilibrium probability-weighted distributions of these features between MSMs.

We find that switch-2 is more likely to adopt closed positions in MYH7b than in β-MyHC (Fig. 6). Crystal soaking experiments have suggested that switch-2 must open in order to allow phosphate release (38). Interestingly, the distances from E469 in switch-2 (human MYH7b numbering) to D464 at the phosphate-binding site and I481 in the relay helix are strongly correlated with the DiffNet output label, suggesting that the position of E469 discriminates the structural ensemble of MYH7b from β-MyHCs (Fig. S5). In the human MYH7b MSM, E469 is more likely to be shifted away from the relay helix, positioning it into the putative phosphate release tunnel, likely blocking phosphate release (Fig. S6). Furthermore, E469 is more likely to adopt positions where its relative position to D464 and I481 is similar to that seen in the IHM free head (Fig. 6*C*). We also note that simulations of MYH7b were more likely to adopt structures where the purine-binding A-loop was in more extended conformations, like that observed in the IHM free head (Fig. S7). Overall, these simulations suggest a structural rationale for the experimental differences in SRX/DRX proportions between MYH7b and β-MYHC.

## Discussion

For decades, the properties of the eight myosin-II isoforms found in mammalian skeletal and cardiac muscle have been a primary focus of the myosin field. Phylogenetic analysis suggests that MYH7b is an ancient sarcomeric myosin that predates the emergence of the skeletal and cardiac-specific myosin isoforms (4). Furthermore, MYH7b orthologs have been found in fish, chicken, snakes, and frogs, suggesting that MYH7b was present in a common vertebrate ancestor (7, 39, 40). Thus, understanding the molecular properties of MYH7b is important and can inform an evolutionary perspective of the sarcomeric myosin family. This myosin gene escaped detection for years because of its unique expression pattern and regulation in mammals that restricts MYH7b protein production to certain striated muscles with specialized functions (5, 7). The surprising discovery of low abundance MYH7b protein in nonmuscle tissue has also sparked recent interest in this myosin's function (10, 41, 42, 43). In contrast to mammals, MYH7b appears to have a role in cardiac and skeletal muscle of birds (7, 11), and as described in this study, pythons. Here, we present the first detailed characterization of the ancient motor properties of sarcomeric myosin MYH7b. We found that although MYH7b has overall slower kinetics and working stroke mechanics than other sarcomeric myosins, the enzymatic properties of MYH7b are consistent with a role in force generation. Furthermore, we identified differential tuning between the motor activities of human and python MYH7b, suggesting evolutionary adaptations based on species-specific physiological demands.

Our results show that several key motor properties of human and python MYH7b are slower than the canonical slow human β-MyHC isoform. Both human and python MYH7b have a reduced actin-activated ATPase activity compared with β-MyHC that is due, in part, to slower kinetics of steps in the chemomechanical cycle. Furthermore, we observe that the sliding velocities of both human and python MYH7b are slower than β-MyHC. In the motility assay, the speed is proportional to the displacement of the working stroke (d) divided by the attachment duration at saturating ATP (*t*_on_). The optical trapping experiments show that the step sizes of MYH7b and β-MyHC are similar. This is consistent with the fact that both β-MyHC and MYH7b have two bound light chains and thus have similar length lever arms. Furthermore, both MYH7b and β-MyHC have two-substep working strokes and thus similar mechanics. Therefore, the differences in the sliding speeds are due to changes in the actomyosin detachment rate, which is limited by the rate of ADP release. Overall, our results suggest that evolutionary pressures have preserved MYH7b activity for slower contractile functions than β-MyHC.

In addition, we observed striking changes in the SRX/DRX ratio of the MYH7b isoforms compared with human β-MyHC. Human MYH7b showed a distinct stabilization of the SRX state, an almost complete reversal of the ratio of SRX/DRX in human β-MyHC. These assays were performed using myosin S1, and our measurements for β-MyHC are consistent with values previously reported for SRX proportions using β-MyHC short S1 and S1 fragments (28, 30, 32). Our experimental results are supported by molecular dynamics simulations, which predict that MYH7b adopts motor domain conformations more similar to those observed in the IHM. Based on these results, we hypothesize that MYH7b may be more likely to stabilize the autoinhibited IHM state. Future work using two-headed constructs with a portion of the myosin rod will be necessary to experimentally confirm this prediction. Stabilization of this ultraslow ATP turnover state could be an evolutionary advantage and energy-saving mechanism as it is known that skeletal muscle myosin can be recruited out of the IHM in situations of increased demand (44, 45).

Given the absence of MYH7b from most mammalian skeletal muscles and cardiac muscle, it appears that these tissues have evolved to use myosin isoforms with faster motor properties. The slow kinetics and high proportion of MYH7b in the SRX state are likely key contributors to the cardiac dilation and dysfunction observed in a transgenic mouse model with forced cardiac expression of MYH7b protein (6). Notably, these results are consistent with a recent study reporting a dilated cardiomyopathy-causing myosin mutation that slows kinetics and stabilizes the SRX state (46). However, specialized mammalian muscles appear to tolerate inclusion of MYH7b; these muscles typically express a broad array of sarcomeric myosin isoforms with different motor properties allowing for functional adaptability (47). For example, the EOM performs both ultrafast movements and slower pursuit and vergence motions (48), which require a repertoire of functionally diverse myosins. The specialized myosin isoform MyHC-extraocular encoded by *MYH13* has the fastest contractile properties of the sarcomeric myosin family and is found exclusively in EOM and laryngeal muscles (3, 49). Likewise, it is possible that MYH7b performs a specialized role in slow contractility in these heterogeneous tissues that show a diverse range of contractility. Finally, MYH7b could copolymerize with other myosins to modulate contractile filaments, specifically in the context of nonmuscle tissues, as has been reported for a distinct class-18 myosin and nonmuscle myosin II (50). Future studies testing the ability to coassemble with nonsarcomeric myosins will be essential for understanding the role of MYH7b in nonmuscle cells and tissues.

In contrast to mammals, which express two cardiac myosin isoforms, pythons express a broad array of myosin isoforms in their hearts and even more in their skeletal muscles. We observed that python MYH7b has slower steady-state actin-activated ATPase activity and slower kinetic rates of key individual steps within the chemomechanical cycle compared with human MYH7b. Intriguingly, despite the lower maximal actin-activated ATPase rate, python MYH7b demonstrated an increase in DRX myosin population compared with human MYH7b. These results suggest that although python MYH7b has overall slower enzyme kinetics, it has been adapted to modulate SRX/DRX such that an increased population of myosin heads are readily available for interactions with actin. The higher proportion of DRX myosin for python MYH7b compared with human MYH7b is potentially the result of evolutionary pressures on body wall skeletal muscles and the heart, which require a constant population of myosin heads in the DRX state to support normal function. This idea is consistent with the requirement for these high-demand muscles to quickly activate in response to environmental cues like predation or threats. In addition, pythons are infrequent feeders that shift between resting, energy-reserving states, periods of high metabolic demand, and postprandial physiological adaptation (51, 52, 53). The slower motor properties of MYH7b and overall significant proportion of myosin in the SRX state could be an evolutionary adaptation to minimize ATP usage during prolonged periods of rest.

In this study, we present the first comprehensive functional characterization of the ancient sarcomeric myosin MYH7b. We have defined the activity of MYH7b as even slower than that of β-MyHC, which was previously the slowest human myosin isoform characterized (2, 13). Our results suggest that MYH7b has been adapted for specialized roles in mammals who do not tolerate the slow properties of MYH7b in conventional skeletal and cardiac muscles. In contrast, the slow properties of MYH7b are likely advantageous to different species like pythons that have different physiological needs and demands on cardiac and skeletal muscles. Questions remain as to whether MYH7b carries out an essential role in mammalian-specialized muscle or whether this myosin is gradually being silenced as is the case in mammalian heart and skeletal muscles. Furthermore, the specific role and mechanism of action of MYH7b in mammalian nonmuscle environments remains unresolved. Ultimately, this study provides a perspective of how highly conserved myosin motors can be differentially tuned for specialized activities across species and tissues.

## Experimental procedures

### RNA isolation, complementary DNA synthesis, and quantitative PCR

Total RNA was purified from Burmese and Ball python ventricle and skeletal muscle by homogenization in Tri Reagent (Molecular Research Center; TR118). Chloroform was added and incubated at room temperature for 15 min followed by a centrifugation spin at 12,000*g* for 15 min at 4 °C. The aqueous layer was removed, and RNA was precipitated with isopropanol and washed with 75% ethanol. RNA pellets were dissolved in HPLC grade water.

RNA was transcribed to complementary DNA (cDNA) using the SuperScript III Reverse Transcription kit (Thermo Fisher/Invitrogen; catalog no.: 18080044) and random hexamer primers. Real-time quantitative polymerase chain reaction was performed on a CFX96-Real-Time PCR Detection System (Bio-Rad) using SYBR Green PCR Master Mix (Thermo Fisher/Applied Biosystems; catalog no.: 4312704) and gene-specific primer sets (Table S3). Relative RNA expression was measured using the Pfaffl standard curve method, and all genes were normalized to 18S expression. Primers were designed against the Burmese python genome (accessed on the National Center for Biotechnology Information), and many primer sets were unable to successfully amplify from RNA produced using Ball python tissues, presumably because of species sequence differences.

### Exon skipping PCR

Amplification of the alternative splicing products was carried out on Burmese and Ball python cardiac and skeletal muscle cDNA by PCR using a forward primer specific to Burmese python exon 5, 5′-TAAGGGTAAGCGGAGGTCT and a reverse primer specific to exon 9, 5′-TTCCATGGCAGGGTTAGC. These primers amplify a 271 base pair product corresponding to the unskipped transcript and a 174 base pair product corresponding to the exon-7 skipped transcript. Primer sequences were checked against unpublished Ball python RNA sequences and designed to match both species.

### Protein lysate preparation and mass spectrometry

Protein lysates were prepared by homogenizing tissue in ice-cold radioimmunoprecipitation assay buffer (50 mM Tris [pH 8], 1 mM EDTA, 0.5 mM EGTA, 0.5% sodium deoxycholate, 1% Triton X-100, 0.1% SDS, 140 mM NaCl with protease inhibitor cocktail [MilliporeSigma/Roche; catalog no.: 11873580001]). Two samples (animals) per tissue type from Burmese pythons and one sample per tissue type for Ball python (because of availability of tissue) were prepared. About 20 μg total protein was run on a NuPAGE 4 to 12% Bis–Tris mini protein gel (Thermo Fisher/Invitrogen; catalog no.: NP0323BOX). The gel was stained with Coomassie blue (Research Products International; catalog no.: B43000) and destained in 50% methanol, 10% acetic acid, and 40% water followed by 10% methanol, 10% acetic acid, and 80% water. Bands at molecular weight marker 250 kDa (MyHC) and between ∼15 and ∼20 kDa (myosin light chains) were excised and sent to the University of Colorado Anschutz Mass Spectrometry Proteomics Shared Resource Facility. Excised protein bands were subjected to tandem mass spectrometry using an Orbitrap Fusion Lumos with Easy nLC 1200 UPLC system (Thermo Fisher).

All MS/MS samples were analyzed using Mascot (Matrix Science; version 2.7.0). Mascot was set to search custom python-specific myosin databases (Burmese python MyHCs and myosin light chains; accession codes are included in Tables S1 and S2) with assumed trypsin digestion. Scaffold (Proteome Software, Inc, version Scaffold_5.1.0) was used to validate MS/MS-based peptide and protein identifications. Peptide identifications were accepted if they could be established at greater than 95% probability by the Peptide Prophet algorithm (54) with Scaffold delta-mass correction. Protein identifications were accepted if they could be established at greater than 99% probability and contained at least two identified peptides. Protein probabilities were assigned by the Protein Prophet algorithm (55). Proteins that contained similar peptides and could not be differentiated based on MS/MS analysis alone were grouped to satisfy the principles of parsimony. Spectrum count normalization is calculated by the Scaffold software by multiplying each spectrum count in each sample by the average count over the biosample’s total spectrum count. A full mass spectrometry sample report including values for percent coverage and number of unique peptides is included in the supporting information (Tables S1 and S2).

### Recombinant myosin expression and purification

#### Construct design

Recombinant human and python myosin was generated by cloning myosin genes into an adenovirus using the pAdEasy Vector system (QBiogene). All experiments used the MyHC motor domain known as S1, which is sufficient to produce the catalytic activity that drives actin-based contractility (56). Myosin S1 constructs correspond to amino acids 1 to 842 for human β-MyHC S1, 1 to 850 for human MYH7b S1, 1 to 850 for python MYH7b S1 followed by a flexible Gly-Ser-Gly or Gly-Ser-Ser linker and a C-terminal 8 amino acid PDZ-binding peptide (Arg-Gly-Ser-Ile-Asp-Thr-Trp-Val). All myosin S1 constructs were expressed in C_2_C_12_ myotubes and purified using the C-terminal tag that binds PDZ domains. The constructs used in these experiments are bound by endogenous mouse C_2_C_12_ light chains (Fig. S2).

The human β-MyHC S1 sequence was obtained by amplifying the S1 region from human heart *MYH7* cDNA as previously described (2). The human MYH7b S1 sequence was obtained by amplifying the S1 region from the human cDNA clone pF1KA1512 (Kazusa DNA Research Institute; product ID: FXC06072). The python MYH7b S1 sequence was obtained by amplifying the gene from Burmese skeletal muscle cDNA using restriction enzyme–carrying primers designed for the 5′ and 3′ regions. All myosin S1 inserts were subcloned into a pUC19 vector containing the PDZ-binding C-tag. Myosin S1 C-tag inserts were subcloned into the pShuttle-cytomegalo virus (CMV) plasmid (Addgene; plasmid #16403), which was transformed into dh5ɑ-competent cells. Colonies were miniprepped and subject to analytical digest to screen for positive clones. The pShuttle-CMV plasmid containing myosin S1 C-tag was transformed into BJ5183-competent cells (RecA+, pAdEasy) and miniprepped. Miniprepped DNA was transformed into dh5ɑ-competent cells and midiprepped to produce pure plasmid sufficient for transfection.

Original pShuttle-CMV constructs were later updated to include the translation enhancing WPRE sequence cloned using a synthetic construct (Genewiz) directly after the C-tag and stop codon using the Gibson HiFi DNA Assembly Cloning Kit (NEB; catalog no.: E5520S). Adenovirus was produced and used to infect C_2_C_12_ cells.

#### Virus production

Replication-deficient recombinant adenoviruses containing myosin S1 were produced by transfecting human embryonic kidney 293 (HEK293) cells and amplified through HEK293 cells to produce adequate quantities of virus. Infected HEK293 cells were collected and lysed by three freeze–thaw steps. Cell debris was pelleted by spinning at 15,000*g* for 30 min, and the supernatant was overlayed onto a CsCl step gradient (1.5 ml of 1.25 g/ml and 1 ml of 1.4 g/ml). Viral particles were separated by spinning at 160,000*g* for 1 h in a Beckman SW 41 Ti rotor. Virus particles were collected, pooled, and purified further using a second gradient of 1.35 g/ml CsCl. Virus was collected and stored at −20 °C in a glycerol-containing buffer.

#### Protein expression

C_2_C_12_ mouse skeletal myoblasts were cultured in Dulbecco's modified Eagle's medium containing 10% fetal bovine serum, 1% l-glutamine, 1% penicillin–streptomycin and differentiated into myotubes using Dulbecco's modified Eagle's medium containing 2% horse serum, 1% l-glutamine, and 1% penicillin–streptomycin. Myotubes were then infected with myosin S1 C-tag adenovirus 3 days after differentiation. Cells were collected 4 days postinfection using trypsin, centrifuged 30 min at 2200*g* at 4 °C, scraped into liquid nitrogen, and stored at −80 °C.

#### Protein purification

Cell pellets were lysed in 50 mM Tris (pH 8.0), 200 mM NaCl, 4 mM MgCl_2_, 0.5% Tween-20, 5 mM DTT, 1 mM ATP, 0.2 mM PMSF, and 1× protease inhibitor cocktail (catalog no.: 11873580001) and mechanically dounced preceding a 25 min centrifugation at 39,000*g*. The supernatant was collected and filtered through 5 and 1.2 μM filters. The filtered supernatant was applied to a column containing SulfoLink resin (Thermo Fisher; catalog no.: 20402) coupled to PDZ. The flow through was collected, and the column was washed with 30 mM Tris (pH 7.5), 50 mM KCl, 5 mM MgCl_2_, 1 mM DTT, and 1 mM ATP. Myosin S1 was eluted using a peptide with higher PDZ specificity (Genescript, Trp-Glu-Thr-Trp-Val). Recombinant myosin-S1 was dialyzed against storage buffer containing 20 mM Mops (pH 7.0), 25 mM KCl, 5 mM MgCl_2_, and 10% sucrose. Proteins were flash frozen after the addition of 1 mM DTT and 1 mM ATP and stored at −80 °C. The resulting purified protein consists of recombinant myosin-S1 with endogenous mouse C_2_C_12_ light chains.

### Actin-activated ATPase assay

Actin-activated ATPase rates were measured using the NADH-coupled ATPase assay. Myosin was thawed on ice and diluted to 0.8 μM in 20 mM Mops (pH 7.0), 25 mM KCl, and 5 mM MgCl_2_ with 5 mM DTT. Rabbit skeletal actin was purified as previously described (57), diluted to reach concentrations ranging from 10 to 100 μM, and was mixed with 4 μM gelsolin (bacterially expressed and prepared from *Escherichia coli* (13)) and incubated for at least 30 min. Myosin was added to actin/gelsolin in a clear 384-well plate to achieve a final myosin concentration of 0.4 μM. One well with myosin alone (0.4 μM) plus buffer was measured to determine the basal ATPase rate in the absence of actin. Right before measuring, 10× buffer consisting of 20 mM ATP, 30 mM phospho(enol)pyruvate (Sigma; catalog no.: 860077), 10 mM NADH (Sigma; catalog no.: N8129), and 8 mM pyruvate kinase/lactate dehydrogenase (Sigma; catalog no.: P0294) was added to each well to achieve a final 1× concentration. Experiments were performed at 25 °C using a SpectraMax iD3 Multimode Microplate Reader (Molecular Devices). Absorbance was monitored at 340 nm every 30 s for 1 h. A standard curve of NADH from 1 to 0.008 mM was run on each plate to determine the conversion factor of nanomoles of NADH/absorbance value.

Individual rates for each myosin and actin well and myosin-alone wells were calculated by taking the linear range of the absorbance *versus* time and converting to nanomoles of ATP/second using the NADH conversion factor. Rates were divided by the myosin concentration in order to obtain the per second rate and plotted against actin concentration. The basal (actin-free) myosin ATPase activity was subtracted from each actin rate, and technical replicates comprised of one or two curves per construct on a given day were averaged. The data were fit to a Michaelis–Menten kinetics equation using the nonlinear fit feature in GraphPad Prism (GraphPad Software, Inc), which calculates the maximum actin-activated ATPase activity rate (*k*_cat_) and Michaelis–Menten constant (*K*_*M*_). Ambiguous fits were discarded across all datasets. Statistical significance was determined using a one-way ANOVA with Tukey’s multiple comparison test.

### *In vitro* motility assay

#### Labeling actin

1 μM F-actin (prepared as described for the actin-activated ATPase assay) diluted in 1× assay buffer (20 mM Mops [pH 7.0], 25 mM KCl, and 5 MgCl_2_) was incubated with 2 μM rhodamine–phalloidin (Thermo Fisher; R415) and incubated 1 h at room temperature. Labeled actin was stored at 4 °C protected from light.

#### Deadheading myosin

A “deadheading” spin down was performed prior to flow cell loading to eliminate inactive myosin bound to actin. A 3:1 M ratio of actin:myosin in 1× assay buffer was incubated on ice for 5 min. 1 mM ATP was added to myosin and actin, and the mixture was centrifuged at 90,000 rpm in a TLA-100 rotor (Beckman) for 25 min at 4 °C. The supernatant was collected and diluted in 1× assay buffer to a final concentration of 0.4 μM.

#### Motility experiments

Flow cells were constructed using a microscope slide, double-sided tape, and coverslips coated in 0.2% nitrocellulose (LADD Research Industries; catalog no.: 53152) diluted in amyl acetate. SNAP-PDZ (plasmid provided by the Spudich Lab (58), purified from *E. coli*) at 3 μM diluted in 1× assay buffer (20 mM Mops [pH 7.0], 25 mM KCl, 5 mM MgCl_2_, and 10 mM DTT) was flowed into each chamber of the flow cell and incubated at room temperature for 2 min. 1 mg/ml bovine serum albumin (BSA) diluted in 1× assay buffer was flowed through each chamber followed by myosin S1 diluted to 0.4 μM (∼54 μg/ml) in 1× assay buffer and incubated at room temperature for 3 min. Each chamber was blocked again with 1 mg/ml BSA followed by labeled actin and incubated at room temperature for 1 min. Finally, motility buffer consisting of 1× assay buffer, 3 mM ATP, 1 mg/ml BSA, 1 mM EGTA, and 0.5% methylcellulose and an oxygen scavenging solution of 4 mg/ml glucose, 0.135 mg/ml glucose oxidase (Sigma; catalog no.: G2133), 0.0215 mg/ml catalase (Sigma; catalog no.: C30). Motility videos were obtained at 25 °C with a frame rate of 1 frame per second for 30 s using a 100× oil objective on a Nikon Ti-E Eclipse Inverted Fluorescence Microscope equipped with a Hamamatsu ORCA-Flash 4.0 V3 Digital CMOS Camera.

#### Analysis of *in vitro* motility data

*In vitro* motility videos were analyzed using MATLAB code developed in-house. Briefly, the code identifies actin fibers between 2 and 6 pixels in width using a Hessian transform (59). A threshold value was used to generate a binary mask of the identified fibers in the image. The resulting mask was then skeletonized, and branches in the skeleton were removed to convert the mask of each fiber into a line. The midpoint coordinate of the line was then identified and used to track the position of the migrating fibers. Each individual fiber was then tracked using a previously developed custom toolbox, which implemented the linear assignment framework (60, 61). The code for this analysis can be accessed at https://github.com/Biofrontiers-ALMC/actin-tracking-toolbox. The mean velocity across technical replicates was determined from the tracked positions, and statistical significance was determined using a one-way ANOVA with Tukey’s multiple comparison test.

### Stopped-flow kinetic experiments

All stopped-flow kinetic experiments were conducted in 25 mM KCl, 20 mM Mops, 5 mM MgCl_2_, and 1 mM DTT, pH 7.0, at 20 °C. A High-Tech Scientific (Kinetic Studios) SF-61 DX2 stopped-flow system was used to perform the measurements, with concentrations stated as those after mixing in the observation cell. All stopped-flow transients were either analyzed in software provided by High-Tech Scientific or GraphPad Prism. Concentrations are those after mixing unless stated otherwise.

Rabbit skeletal actin was purified as previously described (57) and labeled with pyrene as previously described (62). Pyrene-labeled actin was excited at 365 nm using a Hg–Xe lamp, and emission of pyrene-labeled actin was detected after being passed through a KV399 cutoff filter. The binding of myosin S1 to actin quenches the fluorescence signal with dissociation of myosin S1 from actin leading to an increase in fluorescence. As suggested by Fig. S4, it was possible to observe the interactions between actomyosin S1 and ATP or ADP.

The binding of ATP to actomyosin (*K*_1_) is reversible followed by a rate-limiting isomerization of the complex leading the rapid dissociation of the myosin–ATP complex from actin (*k*_+2_). Both ADP and ATP compete to bind to the nucleotide-binding pocket of myosin S1 with ADP binding controlled by the dissociation constant *K*_ADP_ (=*k*_+ADP_/*k*_−ADP_).

The dissociation reaction can be measured using pyrene-labeled actin in complex with S1. The S1 quenches the pyrene label on the actin. When ATP is rapidly mixed with the actomyosin complex, it leads to an increase in fluorescence. Using Fig. S4 and Equation 1, the constants *K*_1_*k*_+2_, *k*_+2_, and 1/*K*_1_ can be determined.

(1) $$k_{obs}=\frac{K_{1}k_{+2}\left[ATP\right]}{1+K_{1}\left[ATP\right]}$$

where *K*_1_*k*_+2_ is the second-order rate constant for ATP binding to actomyosin S1, *k*_+2_ is the maximum rate of ATP-induced dissociation, and 1/*K*_1_ is the ATP affinity for myosin S1.

When ATP and ADP are in rapid competition for binding to the actomyosin complex, Equation 2 can be used to determine the ADP affinity (*K*_ADP_).

(2) $$k_{obs}=\frac{1}{1+\frac{\left[ADP\right]}{K_{ADP}}}$$

The data were graphed in GraphPad Prism. Statistical significance was determined using an unpaired *t* test.

### Optical trapping

#### Protein expression and purification

Cardiac actin was purified from cryoground porcine ventricles as previously described (63). SNAP-PDZ was expressed and purified as described for the motility experiments. Protein concentrations were determined spectroscopically.

#### Optical trapping experiments

Experiments were performed on a custom-built microscope-free dual-beam optical trap, described previously (17). These experiments utilized the three-bead geometry, in which an actin filament is held between two optically trapped beads and brought close to a surface-bound bead that is sparsely coated with myosin (16, 64). All solutions were prepared in KMg25 buffer (60 mM Mops [pH 7.0], 25 mM KCl, 2 mM EGTA, 4 mM MgCl_2_, and 1 mM DTT) unless otherwise specified. Before each experiment, myosin dead heads were removed by ultracentrifugation in the presence of 1 mM ATP and 2.17 μM phalloidin-stabilized actin (436,000*g* 30 min at 4 °C in an Optima MAX-TL ultracentrifuge equipped with a TLA 120.2 rotor [Beckman Coulter]). Rhodamine–phalloidin-stabilized actin filaments (2 μM) were prepared from cardiac actin and 15% biotinylated actin and rhodamine–phalloidin (3.75 μM). Streptavidin beads were blocked in 1 mg/ml BSA by three sequential cycles of suspension in 1 mg/ml BSA followed by centrifugation at 9391*g* for 3 min in a 5424R centrifuge (Eppendorf).

Flow cells were coated sparsely with silica beads suspended in nitrocellulose in amyl acetate, as previously described (17, 64). Each flow cell was loaded with 20 nM PDZ for 5 min, blocked with 1 mg/ml BSA for 5 min, and loaded with C-tagged myosin S1 (20–60 nM) for 5 min. The surface was blocked by washing with 1 mg/ml BSA. The flow cell was then loaded with activation buffer (KMg25 with 1 mg/ml BSA, 1 μM ATP, 192 U/ml glucose oxidase, 48 μg/ml catalase, 1 mg/ml glucose, and ∼25 pM rhodamine–phalloidin-stabilized actin). This was followed by 4 μl of streptavidin beads suspended in 1 mg/ml BSA. The flow cell was then sealed with vacuum grease, and data were collected within 60 min of sealing.

Rhodamine–phalloidin-stabilized actin filaments were attached to polystyrene beads using a biotin–streptavidin linkage, where actin contained 15% biotinylated actin and beads were coated with streptavidin, as previously described (17, 64). For each bead–actin–bead assembly, the trap stiffness was calculated from fitting of the power spectrum as previously described (64). Data were collected at 20 kHz and filtered to 10 kHz according to the Nyquist criterion.

#### Analysis of single-molecule data

All data from optical trapping experiments were analyzed using a custom-built MATLAB (MathWorks) program, SPASM (17). Step size data are reported as mean ± standard error. Statistical testing of the step sizes was done using a two-tailed Student’s *t* test of individual binding interactions. Event durations were fit by single exponential functions using the MATLAB-based program MEMLET (65) to determine the best-fit value for the detachment rate and the associated 95% confidence interval, which was determined by bootstrapping. Statistical testing of detachment rates was performed using a Mann–Whitney test of individual binding interactions.

### Single ATP turnover assay (SRX/DRX)

Single ATP turnover experiments were performed by diluting myosin S1 to 0.8 μM with 1× assay buffer consisting of 30 mM KAc, 10 mM Tris (pH 7.5), 4 mM MgCl_2_, and 1 mM EDTA. Experiments were performed at 25 °C on a CLARIOstar Plate reader with two software-controlled injectors (BMG Labtech). Myosin S1 was added to one well of a 384-well black flat bottom plate (Corning; catalog no.: 3575) to achieve a 0.4 μM final concentration. 2'-(or-3')-*O*-(*N*-methylanthraniloyl) (mant)-ATP, trisodium salt (Thermo Fisher; catalog no.: M12417) was injected into the well at a final concentration of 0.8 μM followed by brief 3 s shake at 300 rpm. At 60 s, excess unlabeled ATP (final concentration of 4 mM) was injected, and fluorescence was monitored every second for 940 s (365 nm excitation/450 nm emission). The fluorescence signal *versus* time was plotted, normalized, and fit to a biexponential decay function using GraphPad Prism to obtain rates and amplitudes of the fast phase (DRX) and slow phase (SRX). Ambiguous fits were excluded across all datasets. Statistical significance was determined using a one-way ANOVA with Tukey’s multiple comparison test.

### Molecular dynamics simulations

We prepared homology models of S1 using the MODELLER software package (66). For the motor domain structural template, we selected a hybrid model of 5N6A with the converter domain of 5N69 because this part of 5N6A was poorly resolved. We followed this approach because both human and python MYH7b motor domains lacked experimental structures. The structural template for the light chain was taken from the light chain in 5N69. For homology modeling of MYH7b, we used the same motor domain and light chain sequences as the construct used in the *in vitro* experiments. For homology modeling of beta-cardiac myosin, we trimmed the motor domain sequence at the same location as Porter *et al.* (34) and used a human ventricular light chain sequence. Homology modeling is summarized in the summary of simulation systems table provided herewith.

### Summary of simulation systems

**Table:** 

Construct	MyHC sequence	Heavy chain structural template	Myosin light chain sequence	Light chain structural template
β-MyHC S1	hMYH7	5N6A/5N69	hMYL3	5N69:H
hMYH7b S1	hMYH7b	5N6A/5N69	MYL1-3f	5N69:H
pMYH7b S1	pMYH7b	5N6A/5N69	MYL1-3f	5N69:H

We prepared systems for simulation in GROMACS (67) following the same procedure as Porter *et al.* (34). The AMBER03 force field and TIP3P water model were used. The protein structures were solvated in a dodecahedral box with a 1 nm pad of TIP3P water. Sodium and chloride ions were added to produce a neutral system at a concentration of 0.1 M NaCl. Each system was minimized using steepest descents until the maximum force on any atom decreased below 1000 kJ/(mol × nm). The system was then equilibrated for 1 ns with heavy atoms placed under a position restraint at 300 K maintained by the Bussi–Parinello thermostat.

Production simulations were then performed on the Folding@Home distributed computing platform using GROMACS 2020 (67). All covalent bonds involving hydrogen were constrained using the LINCS algorithm with a LINCS order of 6 and a LINCS iter of 2 (68). Virtual sites were enabled to allow for a 4 fs timestep (69). An aggregate total of 91.7, 78.5, and 79.0 μs of simulation data was obtained for β-MyHC S1, hMYH7b S1, and pMYH7b S1, respectively (see table of aggregate simulation times and modeling hyperparameters).

#### Markov state modeling

MSMs were constructed by defining microstates using *k*-hybrid clustering with three rounds of refinement with *k*-medoids. A cluster radius of 8.0 Å^2^ was chosen, and the Euclidean distance between residue side-chain solvent-accessible surface area was used as a distance metric as previously described in the study by Porter *et al.* (34). MSMs were fit separately for each isoform by adding a pseudocount of 1/n to each element of the transition counts matrix and row normalizing, as recommended by Zimmerman *et al.* (70). Lag times were chosen by the implied timescales test (see table of aggregate simulation times and modeling hyperparameters and Fig. S8). Important hyperparameters are listed in the table provided below. We find that our results are insensitive to the choice of these hyperparameters.

### Aggregate simulation times and modeling hyperparameters

**Table:** 

Construct	Aggregate simulation time (μs)	Cluster radius (Å^2^)	Lag time (ns)
β-MyHC S1	91.2	8.0	7
hMYH7b S1	78.5	8.0	7
pMYH7b S1	78.9	8.0	7
Total simulation time (μs)	248.6		

#### DiffNets

We employed DiffNets, a self-supervised deep learning framework, to identify structural differences in the active site between the myosin isoform ensembles. We trained a DiffNet on the backbone and C_β_ atoms of the active site. To select active site residues, we chose all residues within 7.5 Å of ADP, Mg, or Pi in the pre-powerstroke crystal structure (Protein Data Bank code: 5N6A). We then converted the atomic coordinates for each isoform’s active site to DiffNets input following the normalization procedure described in the study by Ward *et al*. (36).

We trained this network to learn a latent representation of the active site that could discriminate whether the active site was from either human or python MYH7b or β-MyHC. The initial labels were set to [0,1,1] for β-MyHC, human MYH7b, and python MYH7b, respectively. These labels were iteratively refined as described in the study by Ward *et al*. (36). We selected expectation maximization bounds of [0.1,0.5],[0.5,0.9],[0.5,0.9] for β-MyHC, human MYH7b, and python MYH7b respectively. We used a total of 20 latent variables in the bottleneck layer. The DiffNet was trained for 10 epochs with two hidden layers, 10 epochs with four hidden layers (with the weights of the initial two layers frozen), and 25 “polish” epochs where all weights were tunable. A learning rate of 0.0001 and a batch size of 32 were chosen. All training and analysis were performed using the open-source package available on GitHub at https://github.com/bowman-lab/diffnets.

## Statistical analysis

All data except for optical trapping data and molecular dynamics simulations were graphed and analyzed in GraphPad Prism. Unless otherwise noted, mean ± standard deviation is represented, and *p* < 0.05 was the threshold for significance. Sample size and legend for statistical significance are noted in each figure legend.

## Data availability

All data are included in the article or supporting information. Raw data from this article are available upon request from the corresponding author.

## Supporting information

This article contains supporting information.

## Conflict of interest

The authors declare that they have no conflicts of interest with the contents of this article.
