# Supplementary material for: Functional divergence of the sarcomeric myosin, MYH7b, supports species-specific biological roles
Source: J Biol Chem. 2022 Nov 9;299(1):102657. doi: 10.1016/j.jbc.2022.102657 (PMC9800208; doi:10.1016/j.jbc.2022.102657)
Supplement: Supporting information [file mmc1.pdf]

Supporting Information for

Functional divergence of the sarcomeric myosin, MYH7b, supports species-specific biological roles

Authors

Lindsey A. Lee[1,2], Samantha K. Barrick[3], Artur Meller[4], Jonathan Walklate[5], Jeffrey M. Lotthammer[4], Jian Wei Tay[2], W. Tom Stump[3], Gregory Bowman[4,6], Michael A. Geeves[5], Michael J. Greenberg[3], Leslie A. Leinwand[1,2]

Author Affiliations

[1]Molecular, Cellular, and Developmental Biology Department, Boulder, CO
[2]BioFrontiers Institute, Boulder, CO
[3]Department of Biochemistry and Molecular Biophysics, Washington University School of Medicine, St. Louis, MO
[4]The Center for Science and Engineering of Living Systems, Washington University in St. Louis, St Louis, MO
[5]School of Biosciences, University of Kent, Canterbury, UK
[6]Department of Biochemistry and Biophysics, University of Pennsylvania, Philadelphia, PA.

Supporting Information

This PDF includes:

Figures S1 – S6.

Tables S3 – S7.

Other supporting information for this manuscript include:

Movie S1. Actin sliding motility of human β-MyHC S1. Playback speed 7x.

Movie S2. Actin sliding motility of human MYH7b S1. Playback speed 7x.

Movie S3. Actin sliding motility of python MYH7b S1. Playback speed 7x.

Table S1. Burmese python MS summary. Table lists protein ID probability, percent coverage, exclusive unique peptides, exclusive unique spectrum, exclusive spectrum, total spectrum, and quantitative value for myosin heavy chains (HC) and myosin light chains (LC).

Table S2. Ball python MS summary. Table lists protein ID probability, percent coverage, exclusive unique peptides, exclusive unique spectrum, exclusive spectrum, total spectrum, and quantitative value for myosin heavy chains (HC) and myosin light chains (LC).

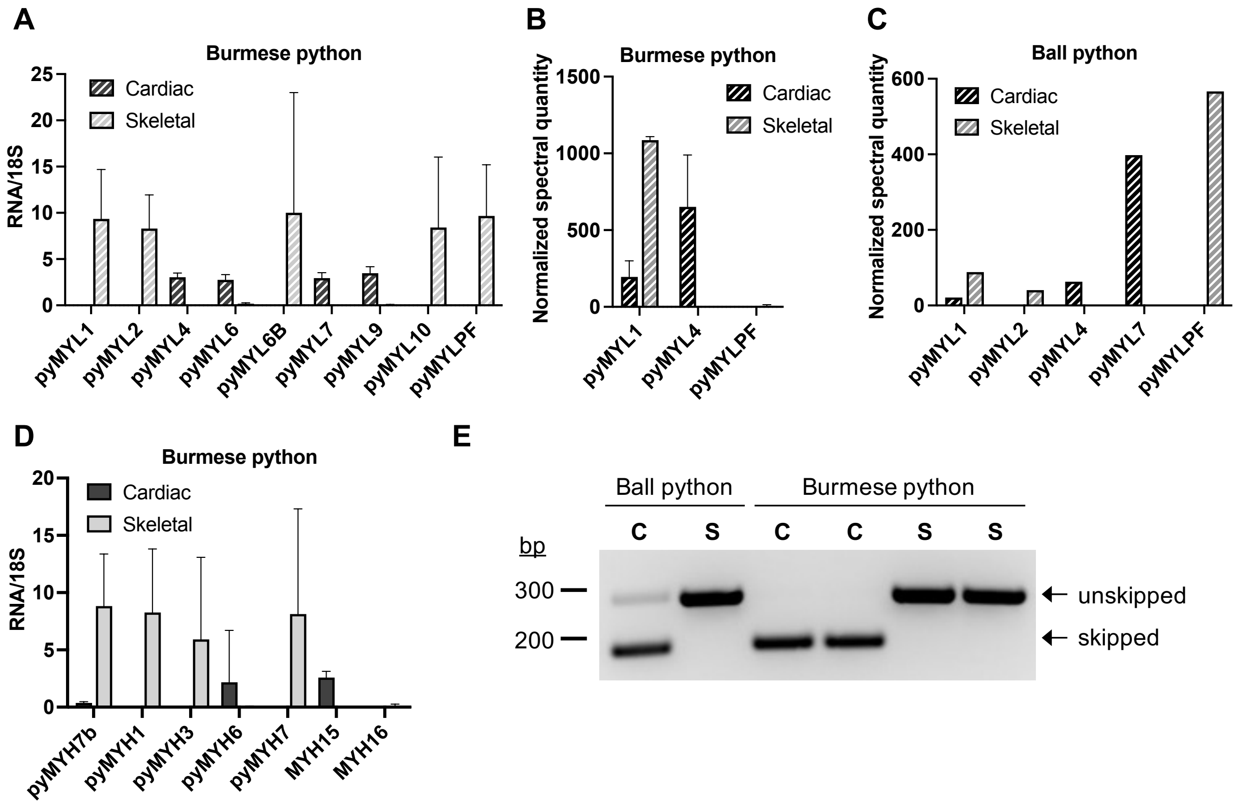

Figure S1. Myosin heavy chain and myosin light chain isoform composition in Burmese and Ball pythons. **A.** Relative RNA expression of Burmese python myosin light chain isoforms in cardiac and skeletal muscle measured by qPCR. **B.** Normalized spectral quantity of myosin light chains in Burmese python cardiac and skeletal muscle measured by mass spectrometry. pyMYLPF was detected in only one skeletal muscle sample at a normalized quantity of 11.4. **C.** Normalized spectral quantity of myosin light chains in Ball python cardiac and skeletal muscle measured by mass spectrometry. Note that without labeled internal standards, values from mass spectrometry are semi-quantitative. **D.** Relative RNA expression of myosin heavy chains in Burmese python cardiac and skeletal muscle measured by qPCR. Data in A-D represent mean ± SD. **E.** RT-PCR analysis of MYH7b exon skipping using a forward primer directed to exon 5 and a reverse primer directed to exon 9, which amplifies exon 7 skipped (174 bp) and unskipped (271 bp) products. C = cardiac, S = Skeletal. Sample sizes for RNA expression analysis: Burmese python cardiac tissue n = 8 and skeletal tissue n=7 and Mass spectrometry: Burmese python n = 2, ball python n = 1. qPCR primers are listed in Table S3.

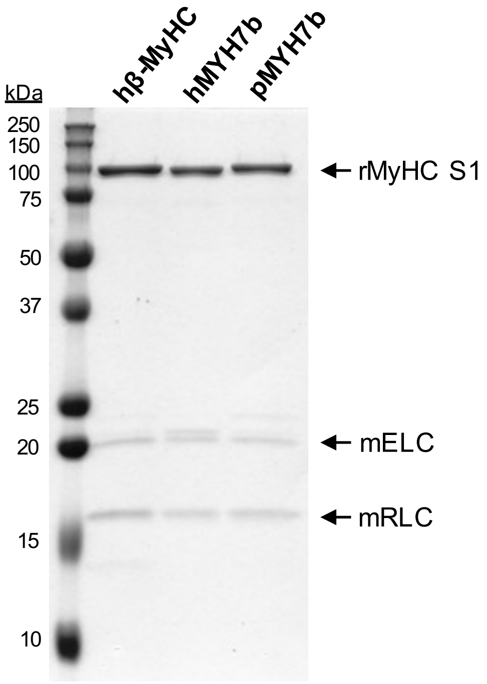

Figure S2. Representative gel of recombinant myosin heavy chain subfragment 1 (rMyHC S1) bound by C_2C_{12} endogenous mouse essential light chain (mELC) and mouse regulatory light chain (mRLC).

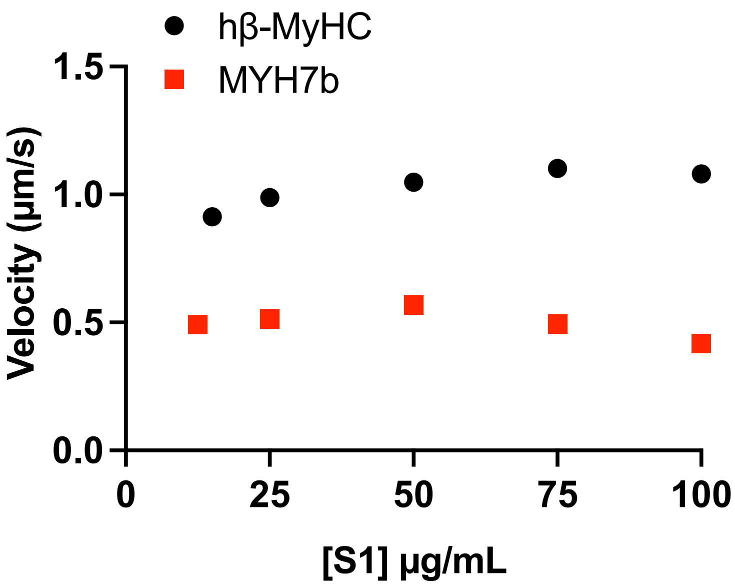

Figure S3. Actin sliding velocity as a function of myosin concentration demonstrate saturation at a concentration of 50 µg/mL myosin S1.

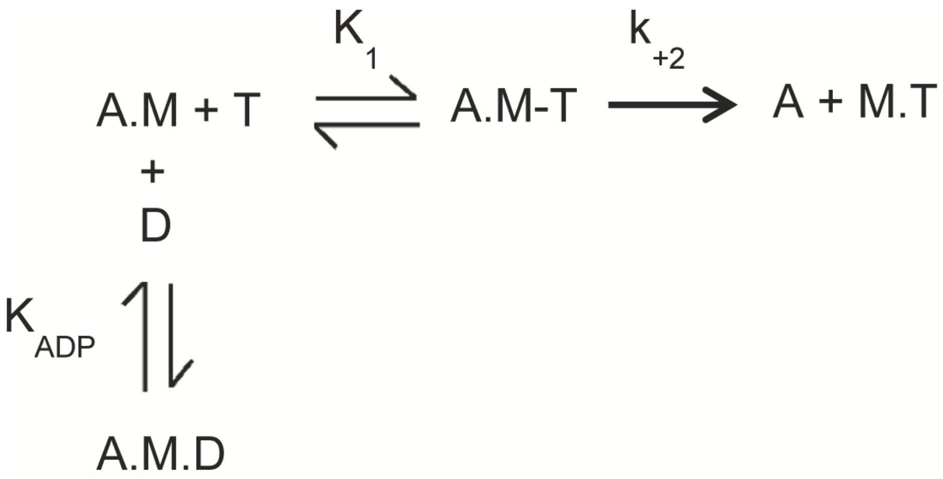

Figure S4. ATP or ADP binding to actomyosin leading to the dissociation of myosin from actin. A, actin; D, ADP; M, myosin/S1; T, ATP.

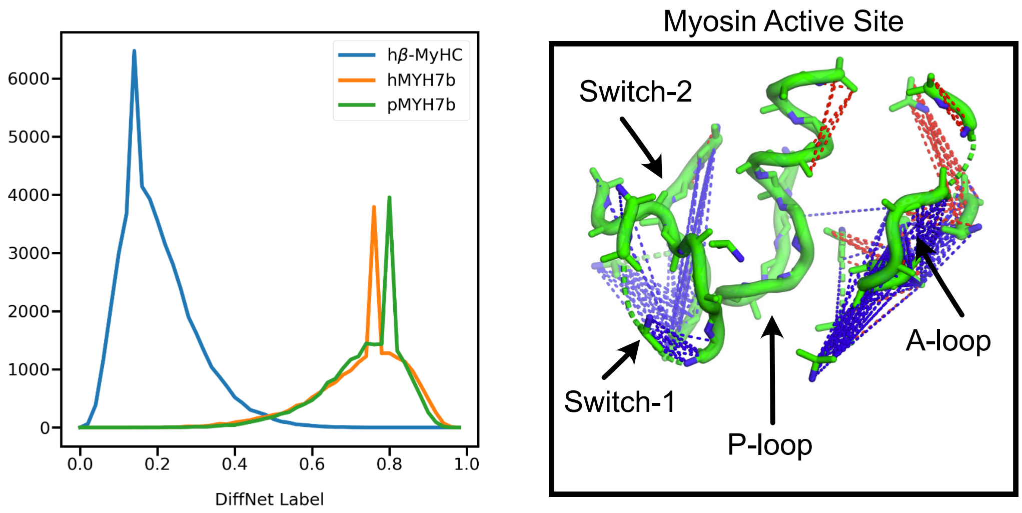

Figure S5. A self-supervised autoencoder called a DiffNet identifies differences between the MYH7b active site structural ensemble and that of β-MyHC. Left: The distribution of predicted labels following training shows that β-MyHC structures generally have small predicted labels while human MYH7b and python MYH7b have larger predicted labels. Right: The DiffNet label is highly correlated with pairwise distances between switch-2 and nearby residues as well as between distances with the purine A-loop. Blue dotted lines indicate distances that tend to be larger in the MYH7b ensembles than in the β-MyHC ensemble. Red dotted lines indicate distances that tend to be smaller in the MYH7b ensembles than in the β-MyHC ensemble.

A

Phosphate Release Intermediate (PDB: 4PJN)

B

MSM Cluster Center

I481-E469 distance: 12.4 Å

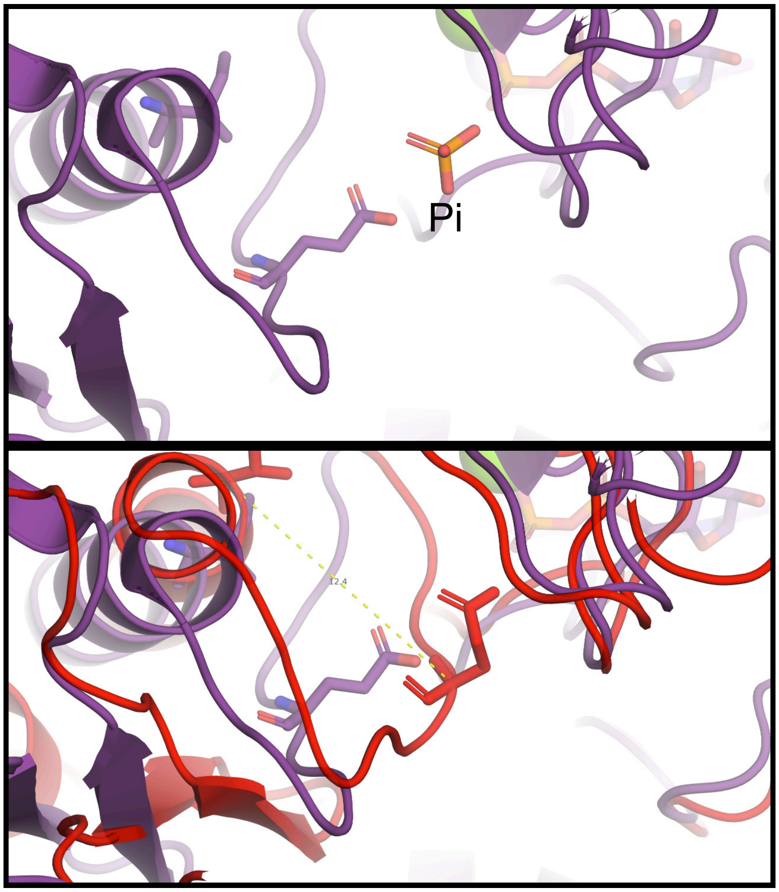

Figure S6. Structural overlay of a human MYH7b cluster center and a crystal structure of a myosin phosphate release intermediate shows that at large I481-E469 distances switch-2 is positioned in the phosphate release tunnel. The sidechain of E469 sterically clashes with the phosphate in the phosphate release intermediate but was removed for visual clarity.

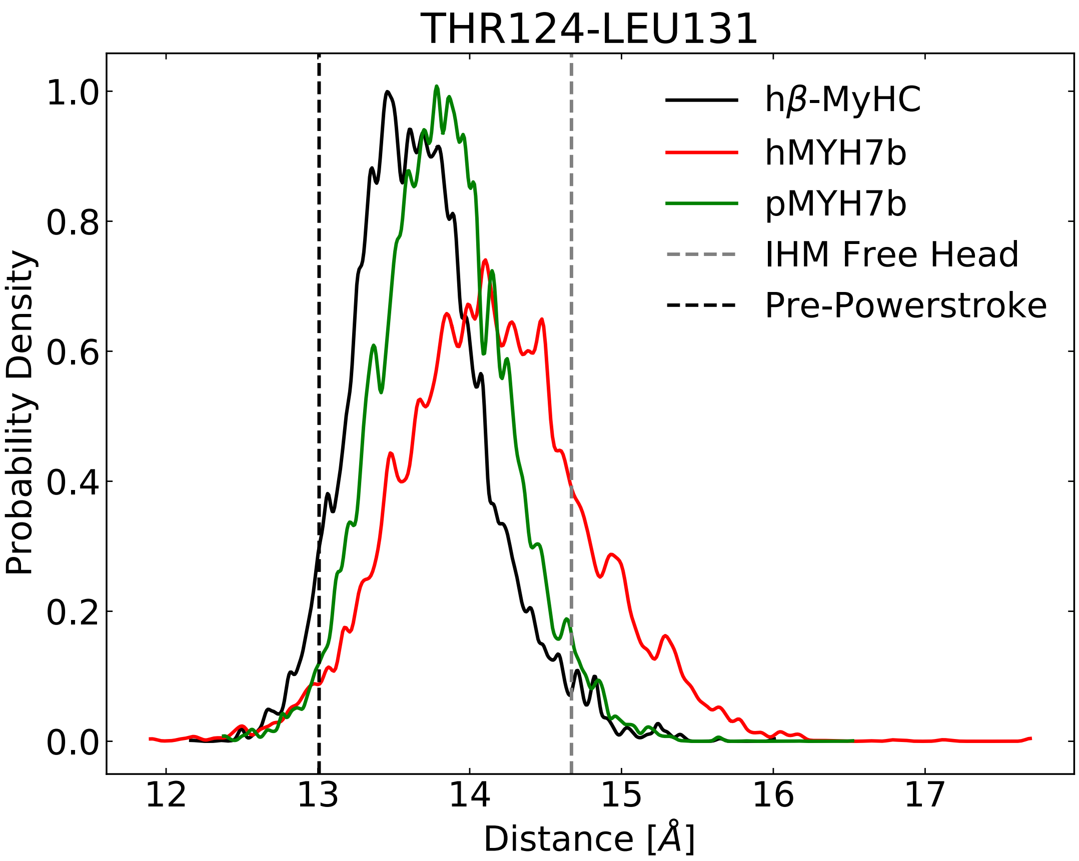

Figure S7. The purine-binding A-loop is more likely to adopt extended conformations in human MYH7b than human β-MyHC as measured by the distance between the C-alpha of T124 and the C-alpha of L131. Dotted lines indicate the value for this distance in a human β-MyHC pre-powerstroke structure (PDB: 5N6A) and the Free Head from the Interacting Heads Motif structure (PDB: 7MF3).

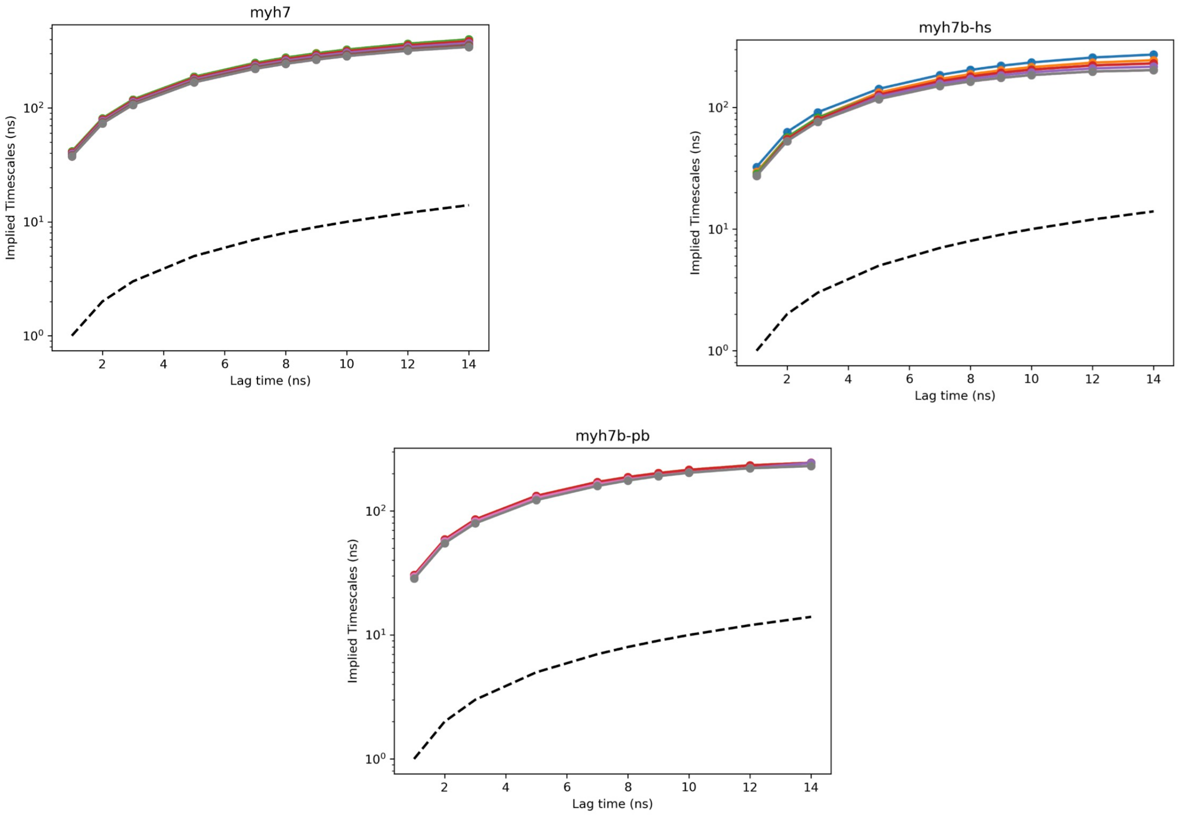

Figure S8. Implied timescales test show logarithmic convergence at ~7 ns across the three isoforms simulated in this study. Myh7 = human β-MyHC, myh7b-hs = human MYH7b, myh7b-pb = python MYH7b.

Table S3. qPCR primers

Primer	Sequence
pyMYL1 F	GTCTTTGACAAGGAGGGCAATG
pyMYL1 R	CTGCCCTTTCATCAGTTCTTCTAC
pyMYL2 F	GATACATTTGCAGCACTAGGACGC
pyMYL2 R	CTTTAAGTTTTTCTCCAAACATTGTCAAGAAC
pyMYL4 F	TTGATCCCAAAAGTGTGACGATTG
pyMYL4 R	TCATCTCTCCAGTTGGGGTCCTGT
pyMYL5 F	GACCCAGAAGCTAAAGGCAATA
pyMYL5 R	GGAGGACTGAAACATCTGATCG
pyMYL6 F	CTCGGAAGATCAGACCGCTGAGTTC
pyMYL6 R	GGCCCTCATCACATCTCCACAC
pyMYL6B F	TCGACCTCTCCAAAGTGGTGATTG
pyMYL6B R	CCTCATCACATCTCCACACTGGC
pyMYL7 F	CTGGCTATGTCAACAAAGATGAGTTTA
pyMYL7 R	CAATGTTTCCAGCCACATCCATG
pyMYL9 F	ACGAGGAGGCCACAGGTTTC
pyMYL9 R	CTTGTCAATTGGAGCTTCTCGATACATCTC
pyMYL10 F	CGGATTCAGGAATTTAAAGAGGCATT
pyMYL10 R	GCTCTTCCATTTTAACATTCATACGGC
pyMYLPF F	TTGGGGAGAAGCTGAAGGGTG
pyMYLPF R	CACTGGGTGGTCAAGAGTTCTTC
pyMYH1 F	GTTAAGAAAGAAGGTGGAGAGTCTGC
pyMYH1 R	GTAGATCATCCAGGCTGCATAAC
pyMYH3 F	GCAAAAAACAGGCATGAAGGGGAC
pyMYH3 R	AAAACGGGAGGAGTTGTCATTCC
pyMYH6 F	GCGTACCAATACATGTTGACAGATCG
pyMYH6 R	CGATACTGGCAAAGTACTGGATAACTCG
pyMYH7 F	GTATCAGCCTTGCACAGGGAGA
pyMYH7 R	CAAAGTGGGGATGGGTGGAA
pyMYH7b F	CTGACCTCATCAAGGGGTTGC
pyMYH7b R	CCTACCGCATACACCACCTGAT
pyMYH15 F	GAGCTGATGGCAACTGATCAAGC
pyMYH15 R	AGTGAGTTTGTAGGCACCGT
pyMYH16 F	GCTGGGCCTGATACTGATCC
pyMYH16 R	ATTTCCTCCTCTTGCCGAGC
py18S F	GCCGCTAGAGGTGAAATTCTTG
py18S R	CTTTCGCTCTGGTCCGTCTT

Table S4. Actin-activated ATPase results summary

Protein	k_{cat} (s^{-1})	k_{cat} SD (s^{-1})	K_M (μM)	K_M SD (μM)	Basal ATPase rate (s^{-1})	Basal ATPase rate SD (s^{-1})	k_{cat}/K_M (s^{-1} μM^{-1})	k_{cat}/K_M SD (s^{-1} μM^{-1})	Biological n (purifications)	Technical n (curves)
hβ-MyHC	1.57	0.18	93.4	56.5	0.110	0.018	0.025	0.015	5	9
hMYH7b	0.80*	0.08	35.9*	9.8	0.109	0.040	0.023	0.006	6	9
pMYH7b	$0.60^{*,‡}$	0.08	79.3	16.1	0.067	0.034	$0.008^{*,‡}$	0.003	4	5

Statistical significance ($p < 0.05$ by one-way ANOVA using Tukey's multiple comparisons test) *compared to hβ-MyHC, ‡compared to hMYH7b.

Table S5. ***in vitro*** **motility results summary**

Protein	Velocity (μm/s)	Velocity SD (μm/s)	Biological n (purifications)	Technical n (movies)	# Filaments
hβ-MyHC	1.017	0.076	3	11	254
hMYH7b	0.597*	0.057	3	12	242
pMYH7b	0.536*	0.050	3	11	178

Statistical significance ($p < 0.05$ by one-way ANOVA using Tukey's multiple comparisons test) *compared to hβ-MyHC.

Table S6. Stopped-flow kinetics results summary

Protein	k_{+2} (s^{-1})	k_{+2} SD (s^{-1})	K_1k_{+2} ($\mu M^{-1}s^{-1}$)	K_1k_{+2} SD ($\mu M^{-1}s^{-1}$)	$1/K_1$ (μM)	$1/K_1$ SD (μM)	K_{ADP} (μM)	K_{ADP} SD (μM)	Technical n (curves)
hMYH7b	520.7	7.0	6.7	1.4	79.8	17.7	120.7	29.5	2
pMYH7b	181.2*	6.9	1.2*	0.0	157.3*	11.5	17.0*	3.2	2

Statistical significance (*$p < 0.05$ by unpaired t-test).

Table S7. Single ATP turnover results summary

Protein	% DRX (fast)	% SRX (slow)	% DRX and SRX SD	k_{fast} rate (s^{-1})	k_{fast} rate SD (s^{-1})	k_{slow} rate (s^{-1})	k_{slow} rate SD (s^{-1})	Biological n (purifications)	Technical n (curves)
hβ-MyHC	85.6	14.4	7.3	0.0604	0.0130	0.00152	0.00078	4	12
hMYH7b	17.1*	82.9*	3.7	0.0361*	0.0171	0.00334*	0.00066	4	10
pMYH7b	53.6*,‡	46.4*,‡	13.3	0.0208*,‡	0.0061	0.00371*	0.00061	5	10

Statistical significance ($p < 0.05$ by one-way ANOVA using Tukey's multiple comparisons test) *compared to hβ-MyHC, ‡compared to hMYH7b.
